# Letter from Prof. Horace Green

**Published:** 1855-05

**Authors:** 


*Letter from Prof. Horace Green.*—We give place, with pleasure, to the following letter from Prof. Green. Perhaps we are taking undue liberty in doing so, as Dr. G. expresses no wish for its publication, but in no other way can we do him entire justice.

It will be seen that our friend explains away many of the objections we offered. The new treatment should rest entirely on its own merits, without regard to preconceived notions of what is possible. If Dr. Green, in the presence of accurate observers, does actually pass his tubes far down into the bronchi, the fact is sufficient without farther argument.

Dr. Green makes no allusion to the experiment we mentioned, proving the impossibility of forcing fluid through a bronchus into a tubercular cavity. We should have added that the cavity, in this case, was lined by a false membrane, which was quite sufficient to account for the result. But as small cavities, where treatment would be most useful, are usually lined by a similar membrane, the experiment has still considerable force.

Dr. G.'s letter is addressed to Dr. Flint in the erroneous supposition that he was the author of the criticism alluded to. H.

12 Clinton Place,
New York, April 7, 1855.

Dr. Flint:

*My Dear Sir,*—I have just received, and have read with much interest—as I do all the numbers of your journal—the one for the present month. For the candor, and the gentlemanly spirit you exhibited, in noticing my paper on catheterism of the air-passages, I thank you. I am so much accustomed to encounter misrepresentation and detraction, among a certain portion of the profession, that it is quite refreshing to have any views, I may advance, received, especially by the profession of my own country, with candor and honorable criticism. Where *this* spirit is manifest, therefore, I am grateful for severe, if just, criticism; and I feel solicitous, under such circumstances—as I do in your case—to remove "objections," and to "satisfy doubts" that may exist, with regard to any statements I may have advanced.

In noticing the experiments with the tube, which you consider as "proving too much," allow me to say that, from a carelessness of phraseology, perhaps, on my part, you have been led to infer, what I did not intend to convey, as having taken place; although, the *facts* occurred as I have stated them. The patient on whom this experiment was performed, is a tall gentleman, of six feet—with a neck rather longer than usual. To introduce the flexible tube the head was raised, and thrown back, in order to bring the open mouth into a line, as near as possible, with the trachea. Of course the neck, by this process, was stretched to its utmost extent. It was in this position the tube was introduced; and, as I have stated, when the patient made "a strong inspiration, the whole instrument, sac and all, was drawn suddenly in, and for a moment disappeared out of sight." But the little sac, which is very thin and delicate, was not drawn into the larynx at all, but was collapsed in the throat of the patient. Nor did the tube all enter the larynx, as a portion of it—from one to two inches—projected above the opening of the glottis. Still, this end of it, as well as the collapsed sac, disappeared for the moment, behind the base of the tongue. Several medical gentlemen *were* present, and saw this operation—although I did not refer to them in connection with this experiment—among whom were my colleague, Prof. Barker, and Drs. J. W. Richards and J. H. Douglas. A few days ago, I passed the same instrument, with ease, into the trachea—belonging to a subject of medium size—a distance of a little over eleven inches from the opening of the glottis—the tube in this instance entering freely, a short way, into a division of the left bronchus; and in the case to which you have referred, the flexible tube, I am confident, was passed into the trachea and bronchial division, from 10½ to 11 inches—the distance which I intended, in my description, to claim.

But, more important than all this, are the questions to which you have referred—the practicability of the operation, its safety, and its effect as a therapeutic agent.

Whatever others may say, or whatever committees may report, I can assure you, the operation of injecting a solution of nitrate of silver into the bronchi, is positively being performed every day in my office, and, in some instances, several times a-day. Its practicability, and its effects, have now been tested on more than sixty patients—among whom are some six or eight physicians—and *in no instance*, have I seen any evidence of injury resulting therefrom. On the contrary, the treatment has proved, more or less, beneficial in nearly every case in which it has been adopted. As I state in my paper, patients laboring under pulmonary disease, particularly those in the early stage of tuberculosis, who have been subjected to this plan of treatment, have, in a large proportion of the cases, continued thus far constantly to improve. Of course it cannot yet be told, in the treatment of phthisis, what the final result will be; I have not claimed for this practice, nor do I propose, until farther trial, to claim for it, in this disease, a "curative value;" although, the results thus far, are exceedingly encouraging. But in asthma, and in chronic bronchitis, particularly in the latter disease, its effects have been, in a high degree, salutary—improvement in every instance (and it has been employed in a large number of cases) has commenced at once, from the use of the remedy. Several physicians are performing this operation, and are employing the treatment with success, as I have learned from various sources. I have to-day received a letter from Dr. M. S. Perry, one of the physicians of Boston Hospital, who writes that he shall attempt the experiment in a few days; and that Dr. Bowditch had already succeeded in three cases. Others will do it, until the practicability and utility of the operation are established or disproved.

I have only to add to this long letter—which has been written hastily, and amid interruptions—that, should you, or any of your personal friends visit our city, it will give me pleasure to have you present at this operation. Your friend, Dr. Lee, had an opportunity of seeing it performed several times, the other day; and, a short time ago, Dr. Mott was also present, and expressed himself perfectly satisfied of its accomplishment.

Very respectfully,

Your obed't servant,

HORACE GREEN.

Austin Flint, M. D.

